# Physicochemical characterization and study of molar mass of industrial gelatins by AsFlFFF-UV/MALS and chemometric approach

**DOI:** 10.1371/journal.pone.0203595

**Published:** 2018-10-09

**Authors:** Simon Duthen, Chloé Rochat, Didier Kleiber, Frederic Violleau, Jean Daydé, Christine Raynaud, Cecile Levasseur-Garcia

**Affiliations:** 1 Laboratoire de Physiologie, Pathologie et Génétique Végétales (PPGV), Université de Toulouse, INP- PURPAN, Toulouse, France; 2 Laboratoire de Chimie Agro-industrielle (LCA), Université de Toulouse, INRA, INPT, INP-Purpan, Toulouse, France; 3 Centre d’Application et de Traitement des Agroressources (CATAR), INPT, Toulouse, France; University of Alberta, CANADA

## Abstract

Industrial gelatins have different physicochemical properties that mainly depend of the raw materials origin and the extraction conditions. These properties are closely related to the molar mass distribution of these gelatins. Several methods exist to characterize molar mass distribution of polymer, including the Asymmetrical Flow Field Flow Fractionation method. The goal of this study is to analyze the relationship between physicochemical properties and the gelatins molar mass distribution obtained by Asymmetrical Flow Field Flow Fractionation. In this study, 49 gelatins samples extracted from pig skin are characterized in terms of gel strength and viscosity and their molar mass distribution are analyzed by Asymmetrical Flow Field Flow Fractionation coupled to an Ultraviolet and Multi Angle Light Scattering detector. This analytical method is an interesting tool for studying, simultaneously, the primary chains and the high-molar-mass fraction corresponding to the polymer chains. Correlation analysis between molar mass distribution data from the different fractions highlights the importance of high molar mass polymer chains to explain the gel strength and viscosity of gelatins. These results are confirmed by an additional chemometric approach based on the UV absorbance of gelatin fractograms to predict gel strength (r^2^Cal = 0.85) and viscosity (r^2^Cal = 0.79).

## Introduction

Gelatin is a protein biopolymer that is widely used in the agri-food industry, medicine (preparation of capsules, treatments for healing), and to a lesser extent in the photographic industry. Gelatin is obtained by extracting collagen fibers by using partial hydrolysis (acidic or basic)[1].

Collagen is the most abundant protein in the animal kingdom (≈30% of all proteins) and is present mainly in the extracellular matrix of various tissues, where it plays a structural role. To date, 27 types of collagen have been identified; they differ from each other mainly in their amino acid composition. Type-I collagen is highly represented because it is used in connective tissue, which is abundant throughout the animal kingdom [2, 3]. The collagen protein is composed of 18 different amino acids and contains a repeated sequence of three amino acids: glycine (26.4%), proline (16.2%), and hydroxyproline (13.5%)[1]. Some of these amino acids, such as proline, have an imine function. The composition of collagen in iminated acids varies according to its origin. When these iminated acids are less represented, the melting temperature of gelatin gels decreases [4, 5]. Mammalian gelatin contains a higher fraction of iminated acid than does fish gelatin, giving mammalian gelatins a higher melting point [6, 7].

The basic unit of collagen is tropocollagen, which is composed of three α chains of about 1050 amino acids, organized in a triple helix that is stabilized mainly by hydrophobic, hydrophilic, or ionic interactions [8]. As per the nomenclature used by Johnston–Banks [9], α represents the chain alone, which has a molar mass of 95×10^3^ g/mol, β represents the double α chain with a molar mass of 190×10^3^ g/mol, and γ represents the triple α chain with a molar mass of 285×10^3^ g/mol.

The main sources of collagen, which are used for the extraction and production of gelatin, are pig skins, bovine skins and bones, and more recently poultry and fish waste [10]. Two varieties of gelatin exist, with the particular variety depending on the type of pretreatment used before extraction. Gelatins that undergo acid pretreatment are referred to by manufacturers as "type A," whereas gelatins that undergo basic pretreatment are referred to as "type B." Each type has different isoelectric points: about 8.5 for type-A gelatins and about 4.5 for type-B gelatins [11]. The combination of pretreatment and hot extraction conditions (pH, duration, and temperature) define the rate of conversion of collagen to gelatin and the physicochemical properties of the resulting gelatin. As a finished product, industrial gelatin gels are characterized by two physical measurements: bloom (a method specifically developed to determine the strength of a gel) and viscosity. These physicochemical properties are closely related to the molar-mass distribution within gelatins [8].

Several methods exist to characterize the molar distribution of polymers. In some cases, the molar masses can be determined qualitatively by using polyacrylamide gel electrophoresis [12]. For gelatin, the molar mass distribution is obtained mainly by size exclusion chromatography (SEC)[8, 13], although this has certain limitations. For example, it does not allow analysis of molecules with a molar mass greater than 7.5×10^7^ g/mol [14], which is problematic for gelatin because it consists partly of aggregates of high molar mass (between 3×10^4^ and 1×10^9^ g/mol)[15]. Thus, SEC cannot cover the entire molar mass range in a single analysis. These aggregates play a significant role in physicochemical properties [16]. Recently, a new method of fractionation, called Asymmetrical Flow Field-flow Fractionation (AsFlFFF) has been developed and allows the analysis of molecules between 1 kDa and a few micrometers. AsFlFFF is now applied in many fields[17]; for example, to study nanoparticles, micelles, emulsions, polymers, and complex proteins [18–22]. In addition, by coupling AsFlFFF with multi-angle light scattering (MALS), the molar mass and radius of gyration of these fragments, which are parameters of interest in the analysis of the molecular distribution, may be determined. For gelatin analysis, the work of Fraunhofer et al.[23] and more recently of Rbii et al.[15, 24] demonstrated that AsFlFFF can be used to fractionate the full range of molar mass in all gelatins, thus enabling the study of high-molar-mass aggregates.

The objective of the present work is to study the relationship between (i) the physicochemical properties, gel strength, and viscosity of gelatin and (ii) the molar distribution obtained by AsFlFFF.

Generally, the ratio of primary chains (α, β, γ) is determined by SEC. However, this technique does not allow the entire range of gelatin molar mass to be studied. The contribution of AsFlFFF is that it can characterize both the molar mass of the primary chains (low molar mass) and that of the aggregates (high molar mass). Colloids with high-molar-mass fragments have a higher viscosity [25] for a given concentration. Because AsFlFFF covers these high molar masses, it provides a tool that is more precise than SEC for understanding the viscosity. In addition, it allows us to determine the gel strength.

The contribution of this study is thus to characterize simultaneously both primary chains and high-molar-mass aggregates in gelatins. Although chromatograms obtained by SEC are traditionally studied by integrating the chromatographic peaks, the approach proposed herein is based on applying datamining techniques to infrared spectra [26, 27]. The aim is not to use individual peaks, but to use the entire fractogram (obtained from AsFlFFF).

## Materials and methods

### Gelatin samples

This study used 49 samples of industrial gelatin produced in a French gelatin dish, in 2016. Type-A gelatins (acidic extraction), extracted from pig skins, were selected to cover the ranges of gel strength (0–350 Bloom) and viscosity (0–0.01 Pa.s) commonly found in gelatins (see Table 1).

**Table 1:** Physico-chemical properties of sampled gelatins: Number of samples n = 49, mean, standard deviation (SD), minimum and maximum and coefficient of variation (CV) of gel strength, and gelatin viscosity.

	Gel strength	Viscosity
**Mean**	231.93 g	0.00397 Pa.s
**Min**	45.05 g	0.001517 Pa.s
**Max**	323.23 g	0.00977 Pa.s
**SD**	75.41 g	0.001562 Pa.s
**CV**	32.51%	39.34%

### Physico-chemical analysis

#### Gel strength

Gel strength was determined by using the Bloom method, which corresponds to the force required by a 12.7-mm-diameter piston to push 4 mm deep into a gelatin gel. A 6.67% aqueous gelatin solution was prepared in 60-mm-high, 59-mm-inner-diameter glasses suitable for measurement (SCOTT, 2.102.501). A gelatin solution was prepared by diluting 7.5±0.1 g of gelatin in 105 ml of distilled water (Milli-Q, Millipore, Bedford, MA, USA) at 20°C. The glasses were placed in a first bath at 40±2°C for 10 minutes, then transferred to a second water bath at 65±2°C for 20 minutes to allow the gelatin to completely dissolve. The glasses are finally placed in a bath at 10±0.1°C for 17±1 h, leading to gelation. The gel strength (or Bloom value) was measured directly by using a gelometer (Stevens-LFRA, C. Stevens and Son, Ltd., United Kingdom), which determines the maximum force required for the gelometer cylinder to penetrate 4 mm into the gel at a speed of 0.5 mm/s.

#### Viscosity

The gelatin viscosity was defined by the time required for a continuous flow of a given volume of gelatin in solution at a given temperature. This time was then converted to estimate the viscosity [28] (NF EN 12092, August 2002). For this purpose, a 100 mL viscometer pipette (with an upper and lower gauge line) calibrated at 60°C was placed vertically in a circulating immersion heater set at 60±0.1°C. Gelatin solutions at 6.67% (w/w) were prepared by dissolving 7.5±0.1 g of gelatin in 105 ml of distilled water at 20°C (Milli-Q, Millipore, Bedford, MA, USA). The swelling and solubilization steps were identical to those described above for the gel strength (Bloom).

Immediately after solubilization, the gelatin gel was transferred to the viscometer pipette and the measurements were repeated. The analysis began when the gelatin reached 61°C, and the viscosity was determined by the flow time.

### Analysis of molar distribution by AsFlFFF-UV/MALS

#### Eluent

The eluent was produced by using the Fraunhofer method [23], which involves preparing a solution of 2 mM sodium phosphate and 14 mM sodium chloride. The pH of the solution was adjusted to 6 by adding a phosphoric acid solution (85% v/v). The eluent was then filtered through 10 and 40 μm polytetrafluoroethylene diaphragms (Pall Corporation, Ann Arbor, MI, USA) by using a vacuum pump (Vacuum/Pressure Pump, Pall Corporation, Ann Arbor, MI, USA).

#### Preparation of gelatin solutions

The gelatin was solubilized at 3.3% (w/w) by removing 1.66 g of gelatin for 50 mL of distilled water (Milli-Q, Millipore, Bedford, MA, USA). The tubes were then placed in a heated stirrer (MultiTherm Shaker, Benchmark Scientific Inc, NJ, USA) at 37°C and stirred at 500 rpm for 12 h, then centrifuged (Centrifuge MR 23i, JOUAN SA, St. Herblain, France) at 13 000*g*, at a temperature of 37°C for 5 minutes. After centrifugation, 0.605 mL of supernatant was extracted and diluted with eluent to obtain a 0.2% (w/w) solution. From this solution, triplicate samples were prepared for analysis by AsFlFFF-UV/MALS, as described below.

#### Analysis by asymmetrical flow field-flow fractionation

The AsFlFFF analysis was done by using a Dual Tech System (Wyatt Technology Europe, Dernbach, Germany) coupled with a Dionex® ultimate 3000 Series high-performance liquid chromatography (HPLC) system (LC-Packings, Dionex, Amsterdam, The Netherlands) connected to an 18 angle multi-angle light scattering (MALS) Heleos II instrument (Wyatt Technology, Santa Barbara, CA, USA) and an ultraviolet (UV) detector The MALS detector used a laser with a wavelength of 690 nm, and the detector was calibrated by using a standard Bovin Serum Albumin (BSA). The intensity of the light scattering was calibrated by using a pure toluene solution (HPLC quality). The samples were injected into the AsFlFFF channel by the HPLC system equipped with an in-line vacuum deaerator. A 0.1 μ m in-line filter (VVLP, Millipore, Germany) was placed between the pump and the channel. The AsFlFFF channel has a trapezoidal geometric shape; it was 19.5 cm long, 1.65 cm wide at the cell entrance, and 0.27 cm wide at the cell exit. Inside this channel, the thickness was provided by a 250 μm Mylar film placed on an ultrafiltration membrane with a molar mass cut-off of 5 kDa (Wyatt Technology Europe, Dernbach, Germany). The samples were analyzed in triplicate.

The conditions were as follows for elution with AsFlFFF: During the focus-injection stage, the eluent was injected through the inlet and outlet of the channel and eliminated through the membrane. The cross flow was then fixed at 1.5 mL/min for one minute. Next, 30 μL of the sample to be analyzed were injected at a flow rate of 0.2 mL/min for four minutes, with the cross flow maintained at 1.5 mL/min.

At the end of the focus-injection stage, the outflow from the channel toward the detectors was fixed at 1 mL/min. At the beginning of the elution phase, the cross flow was fixed at 4 mL/min for four minutes, then the flow was reduced linearly over 20 min from 4 mL/min to 0.1 mL/min. Finally, the flow was held constant at 0.1 mL/min until the end of the analysis.

Following the work of Tromp et al. [8] and Rbii et al.[15], for gelatin, a refractive index increment *dn*/*dc* of 0.164 mL/g and an UV molar extinction coefficient *Ɛ* = 12 700 mL g^−1^ cm^−1^ were used to calculate the molar mass (number molar mass Mn and number average mass Mn). These same parameters were also used to calculate the gyration radius (Zimm (30)) (ASTRA soft version 6.1.1.17, Wyatt technology, Santa Barbara, CA, USA).

The radius of gyration can be considered as the distance from an axis to a virtual point mass so that rotational inertia is the same as that of the molecule. The number average mass is the sum of the molar mass of the polymers present, weighted by the number of polymer chains of each mass, whereas the weight-average molar mass is the average of the molar mass weighted by the mass of a chain of each length.

#### Analysis of UV absorbances of gelatin fractograms

The chemometric processing of the data was done by using The Unscrambler® v. 10.3 (CAMO software AS, Oslo, Norway). The UV absorbencies measured every 0.49 s during the elution from 10 to 40 min constitute the 3665 variables. The average fractogram of the three repetitions of the samples was considered for the 49 samples analyzed, so the resulting matrix was of type *n*×*t* with *n* = 49 and *t* = 3665.

The Bloom gel strength and the viscosity-prediction models were developed by partial least squares (PLS) regression. The models were developed in cross validation. The quality of the calibration models was evaluated by looking for the highest coefficient of determination between predicted and measured values in calibration and cross validation (r^2^cal and r^2^cv) and the lowest residual variance (root mean square error) in calibration and cross validation (RMSEC and RMSECV)[29].

## Results

### Characterization of molar distribution of gelatins

**Fig 1:**
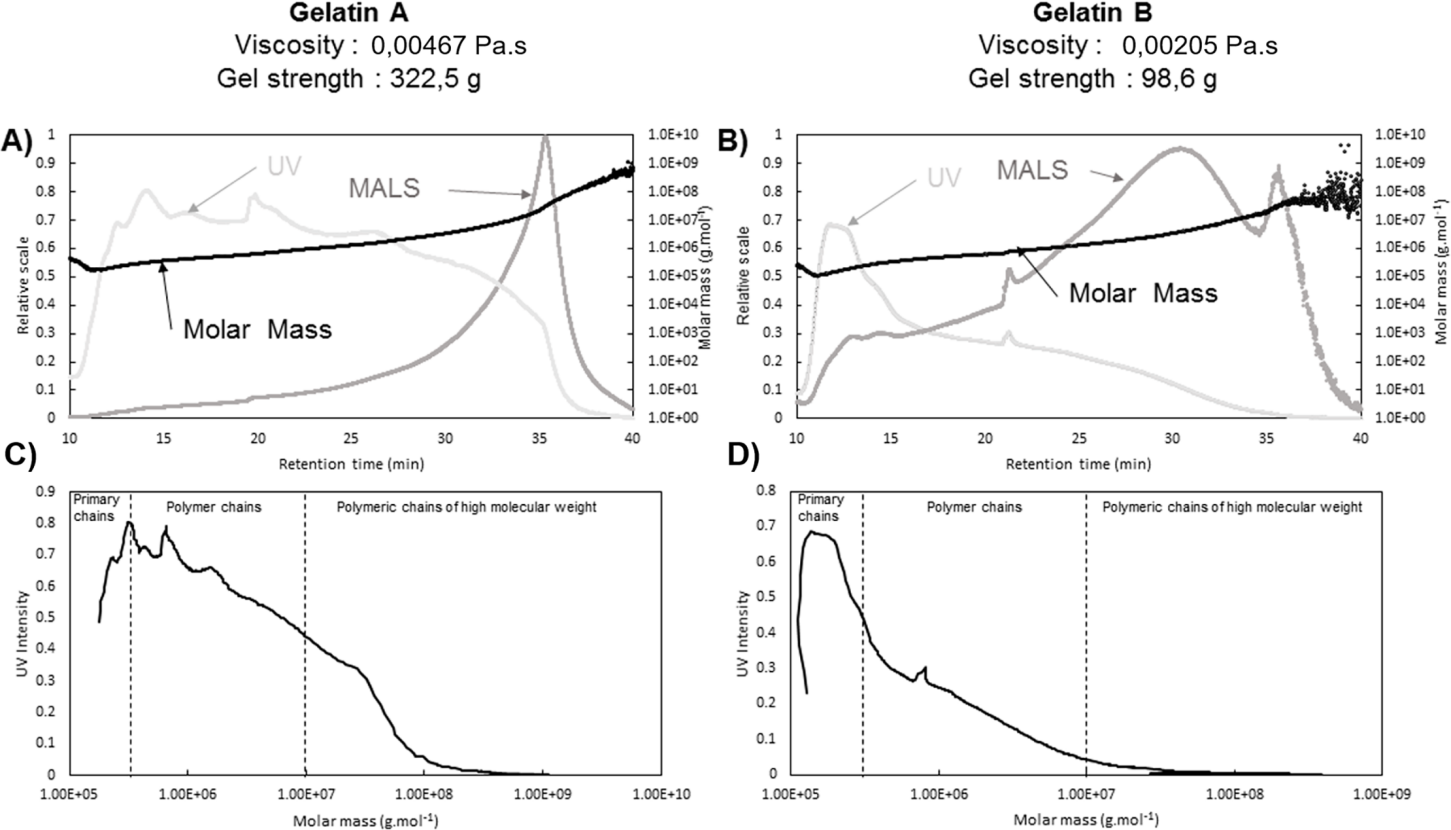
Panels A and B show the fractograms from AsFlFFF-UV/MALS analysis of pork gelatins A and B as a function of elution time. Panels C and D show the UV absorbance as a function of molar mass. Gelatin (A, C) has a gel strength of 322.46 g and a viscosity of 0.004671 Pa.s, gelatin (B, D) has a gel strength of 98.64 g and a viscosity of 0.002054 Pa.s. In panels A and B, the relative UV absorbance is shown in light gray and the relative intensity of the 90° light scattering signal is shown in dark gray. The abscissas of both are the left axes. The molar mass in g/mol is shown by the black dots in panels A and B (right axes).

Fig 1 shows two fractograms that are characteristic of two gelatins with very different technological parameters: a high-viscosity gelatin A (0.004671 Pa.s) with high gel strength (322.46 g), and a low-viscosity gelatin B (0.002054 Pa.s) with low gel strength (98.64 g).

The UV intensity corresponds quantitatively to the presence of the protein, and the MALS signal corresponds to the light scattering (at an angle of 90° in Fig 1A and 1B) and is a function of molar size. For polymers of the same type, the MALS signal was used to calculate the molar mass, the increase of which, considering the analysis conditions, was almost linear relationship throughout the elution. Ultimately, the combination of UV absorbance and MALS provides a global method to obtain the distribution of gelatin fraction as a function of molar mass. The molar mass is found to increase steadily during elution and attains 1×10^9^ g/mol.

The two gelatins A and B do not have the same quantitative distribution of protein polymers throughout elution. The UV intensity of gelatin A ranges from 0.4 to 0.8 from the 10th to the 35th minute of elution, indicating a homogeneous quantitative distribution of polymers over the entire range of molar masses. The same is not true for gelatin B: at the 15th minute of elution, the UV absorbance is less than 0.3 and gradually decreases until the 35th minute, which indicates the presence of a small fraction of polymers of mass ranging from 4×10^5^ to 5×10^8^ g/ mol.

We thus decided to separate the diagrams into two distinct sequences (Fig 1C and 1D): the low-molar-mass fraction corresponding to the primary chains and the high-molar-mass fraction corresponding to the polymer chains. The primary-chain fraction consists of the α chains of the gelatin protein of molar mass ranging from 0.5×10^5^ to 1×10^5^ g/mol, the β dimer with molar mass ranging from 1×10^5^ to 2×10^5^ g/mol, and the γ trimer with molar mass ranging from 2×10^5^ to 4×10^5^ g/mol [15]. The primary-chain fraction contains molar masses from 0.5×10^5^ to 4×10^5^ g/mol. The total fraction represents all primary chains and polymer chains, covering a range from 0.5×10^5^ to 1×10^9^ g/mol. Within the protein fraction, polymers with molar mass exceeding 1×10^7^ g/mol and polymers with smaller molar mass are differentiated. In fact, the fraction of polymers of higher molar mass constitutes a differentiating characteristic for the gelatins studied (Fig 1C and 1D).

### Radius of gyration of gelatin polymers

**Fig 2:**
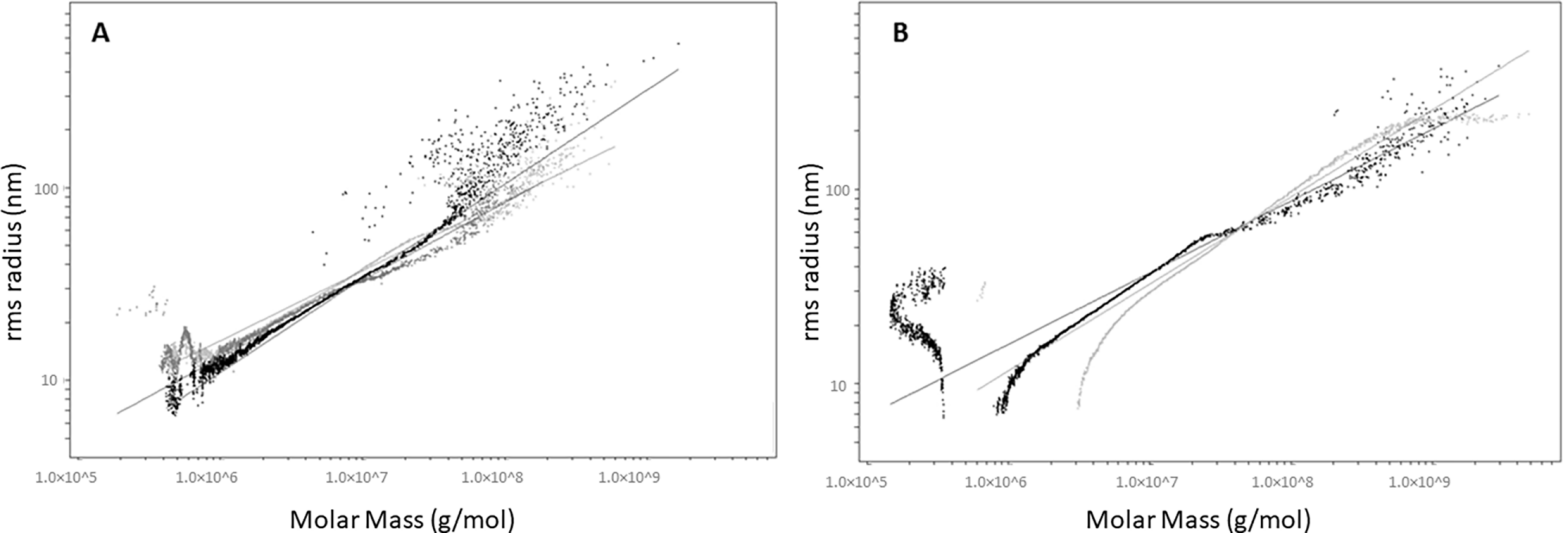
RMS conformation plot for (a) low- and medium-bloom gelatins and (b) for high-bloom gelatin. In panel (a), the three gelatin types are 200 g gel strength and 0.00352 Pa.s viscosity (light gray), 97 g gel strength and 0.00202 Pa.s viscosity (dark gray), and 214 g gel strength and 0.00565 Pa.s viscosity (black). Panel (b) shows two gelatins, gel strength 268 g and viscosity 0.01069 Pa.s (light gray) and gel strength 214 g and viscosity 0.00567 Pa.s (black).

The analysis of light scattering during elution yields the radius of gyration of the separated molecules. Fig 2 shows the root means square (rms) radius of gyration as a function of molar mass on a log-log scale. Below 1×10^6^ g/mol and above 1×10^9^g/mol, data are not interpretable. According to the light-scattering principle of Wyatt and Zimm [30, 31], the light scattering associated to a molecule diffuses anisotropically if its diameter is less than 1/20th of the wavelength of the incident monochromatic light. This renders the measurement of the radius gyration unreliable. When irradiating with a laser at 690 nm, the estimated radius of gyration should be about 15 nm for molecules of a regular conformation.

The data presented in Fig 2 show that, for a radius of gyration less than 15 nm or below 1×10^6^ g/mol, the results are no longer interpretable. These results show that, for molar mass greater than 1×10^6^ g/mol, the radius of gyration as a function of molar mass is independent of the gelatin considered (slopes are between 0.35 and 0.50 nm mol/g). For Fig 2, the gelatins were selected to cover the full range of molar mass up to 1×10^9^ g/mol, which corresponds to a maximum radius of gyration of 250 nm.

Fig 2A shows gelatins with low physicochemical properties (low viscosity and low gel strength), whereas Fig 2B shows two gelatins with high physicochemical properties (high viscosity and high gel strength). Although the slopes of the different types of gelatins are similar, the range of molar mass contain in each gelatin is not the same. According to the work of Wyatt [31], the slopes observed in Fig 2 should be characteristic of a polymer with a conformation intermediate between a fibrillar structure (slope ≥ 0.5) and a globular structure (slope ≤ 0.33). The gelatins studied herein present molar distributions that fall into the range of various polymers, probably with the same structure, because the radius of gyration as a function of molar mass is similar for all gelatins.

The results for the radius of gyration indicate that gelatins, whatever they may be, are similar to each other and have a conformation intermediate between a globular and a fibrillar structure. Conversely, when going from gelatin with high gel strength to one with low gel strength, the ratio of radius of gyration to molar mass remains the same, although the data cloud may shift horizontally on the graph. Finally, although the molar masses vary between the various gelatins, this ratio remains the same, which means that the range of molar mass varies for different gelatins. For each gelatin, the maximum molar mass and the maximum radius of gyration is specific to the given gelatin and is related to its physicochemical properties.

### Variation in molar distribution of gelatins

**Fig 3:**
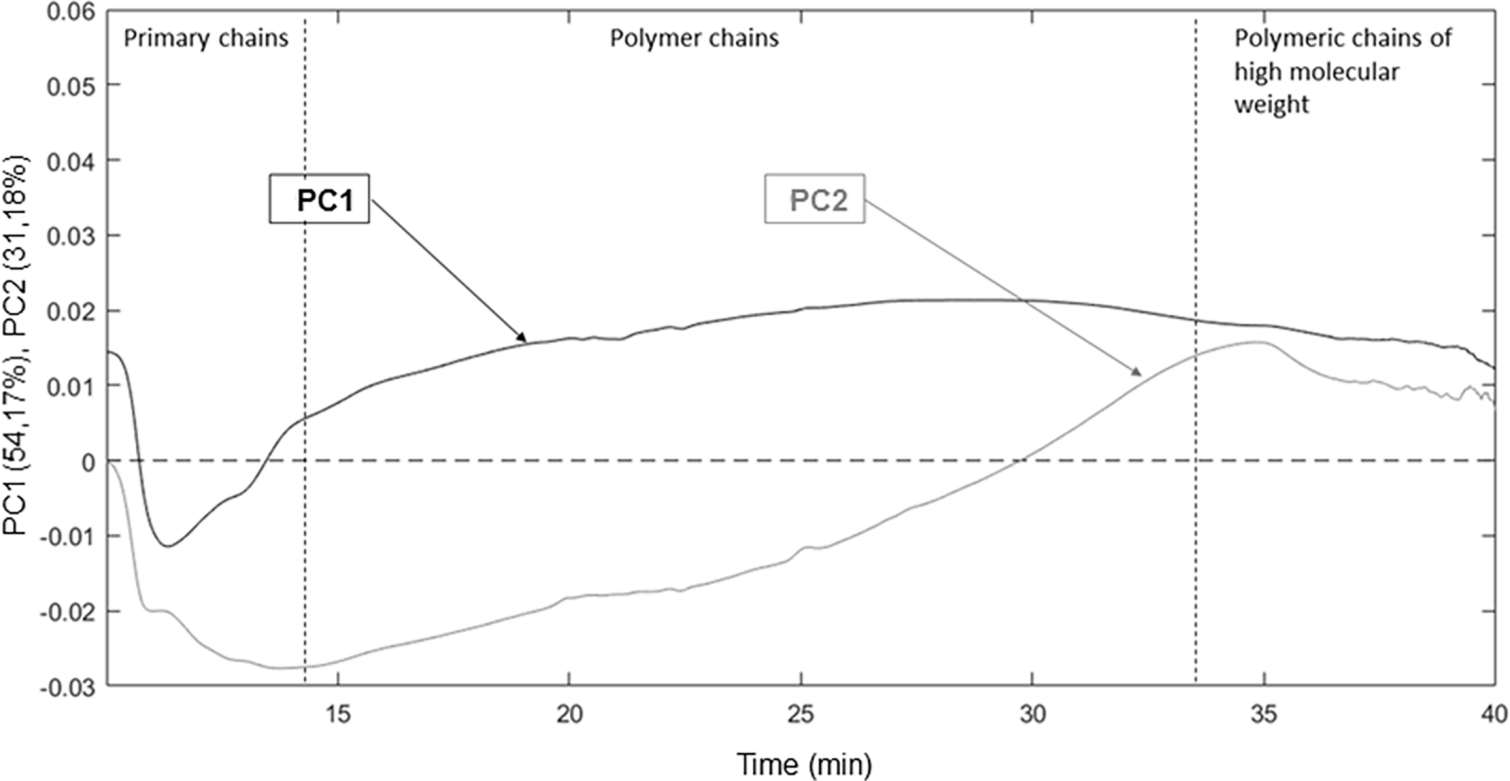
Contribution by main components of UV absorbance as a function of elution time. CP1 (CP2) is shown in black (gray).

Fig 3 shows the first two components (PC1 and PC2) of a principal component analysis of the UV absorbance of the 49 gelatin samples as a function of elution time. PCA is an orthogonal transformation that converts the set of possibly correlated infrared variables into a set of linearly uncorrelated variables called principal components (PCs). PCA is defined so that the first PC accounts for as much variability in the infrared spectra as possible, with the subsequent PCs accounting for less and less variability. These main components represent the sources of variability in the data.

The first three components account for 90.13% of the variation in the UV absorbance between gelatins, with CP1 alone accounting for 53.71%. The absorbance of low-molar-mass molecules, eluted between 10 and 15 minutes, appears to contribute little to CP1. However, CP1 increases gradually with elution time (i.e., as molar mass increases).

CP2 accounts for 31.76% of the variation in the UV absorbance. A negative correlation exists with low-molar-mass molecules and a positive correlation exists with high-molar-mass molecules (30 to 40 min). This component reflects in particular the contribution of the primary-chain fraction and the decreasing contribution of low-molar-mass polymeric chains (<10^7^ g/mol). Conversely, a positive contribution exists with the fraction of very-high-molar-mass polymer chains (>5.10^7^ g/mol).

Note that the model of gel strength and viscosity is not reflected in a specific area of the fractogram (i.e., by specific range of molar mass). Instead, the model is reflected by the overall fractogram (i.e., all primary chains and aggregates). Thus, molecules of all size influence the physicochemical properties, so the global chemometric approach used here allows the fractogram data to be interpreted differently than for a classical study of the distribution of molar mass.

These data reflect the two major sources of variation in the distribution of molar mass for the gelatin samples analyzed. The first and most important is the variation in the range of gelatin polymers (in the molar-mass range from 4.10^5^ to 5.10^7^ g/mol). The second is the clear opposition between gelatin samples that contain a high fraction of primary chains and those with a high fraction of high-molar-mass polymeric chains.

### Correlations between gel strength, viscosity, and molar mass of AsFlFFF fractions

Table 2 presents the similarity matrix between gel strength, gelatin viscosity, Mn, Mw, and the mass fractions for primary chains and polymer chains.

Previous work on this subject is not conclusive. In fact, several publications explain the variation in physicochemical properties in terms of amino-acid composition, raw material, or the nature of the process[1, 7, 32, 33]. In addition, most previous work focuses on primary chains to study the physicochemical properties [8, 14].

**Table 2:** Similarity matrix (Pearson correlation coefficient) between gel strength, viscosity, Mn, Mw, and mass fraction for primary chain fractions (PRI) and polymer chains (POLY) fractions for 49 samples.

	Bloom	Viscosity	Mass fraction PRI (%)	Mass fraction POLY (%)	Mn PRI (kDa)	Mw PRI (kDa)	Mn POLY (kDa)	Mw POLY (kDa)
**Bloom**	1							
**Viscosity**	0.613	1						
**Mass fraction PRI (%)**	−0.604	−0.429	1					
**Mass fraction POLY (%)**	0.503	0.325	−0.935	1				
**Mn PRI (kDa)**	**0.556**	**0.303**	−0.780	0.780	1			
**Mw PRI (kDa)**	**0.535**	**0.251**	−0.780	0.822	**0.976**	1		
**Mn POLY (kDa)**	**0.625**	**0.645**	−0.758	0.148	0.167	0.113	1	
**Mw POLY (kDa)**	**0.099**	**0.341**	−0.748	−0.003	0.128	0.126	**0.284**	1

The primary-chain fraction is negatively correlated to gel strength and viscosity. Conversely, the polymeric-chain fraction is positively correlated to the physicochemical properties. The viscosity is less strongly correlated to each of these fractions than is the gel strength.

For the primary-chain fraction, Mn and Mw are strongly correlated (*R* = 0.976), which shows that the 49 samples are identical in terms of primary-chain composition. In contrast, for the polymer-chain fraction, Mn and Mw are not correlated (*R* = 0.284). In fact, for two different samples with similar Mn and very different Mw, the composition in molar mass is very different. The 49 gelatins therefore differ in their composition of high-molar-mass aggregates. The distribution of high-molar-mass polymers therefore greatly contributes to the differentiation of gelatins.

The gel strength tends to correlate with Mn and Mw for the primary chains (*R* > 0.5). However, for the primary chains, the viscosity is not correlated with Mn and Mw (*R* ≤ 0.3). Both the gel strength and viscosity correlate with Mn for the polymer-chain fraction (*R* = 0.625 and 0.645, respectively) but not with Mw (*R* = 0.099 and 0.341, respectively). Polymer chains therefore play a key role in determining bloom and viscosity. The value of Mw is much more sensitive to extreme values than is the value of Mn, so Mw is less reliable than Mn.

These results support the privileged role played by high-molar-mass polymeric fractions in the expression of gel strength and gelatin viscosity. Conversely, a high fraction of primary chains would be unfavorable to the expression of gel strength and viscosity.

### Prediction of gel strength and viscosity from molar distribution

**Fig 4:**
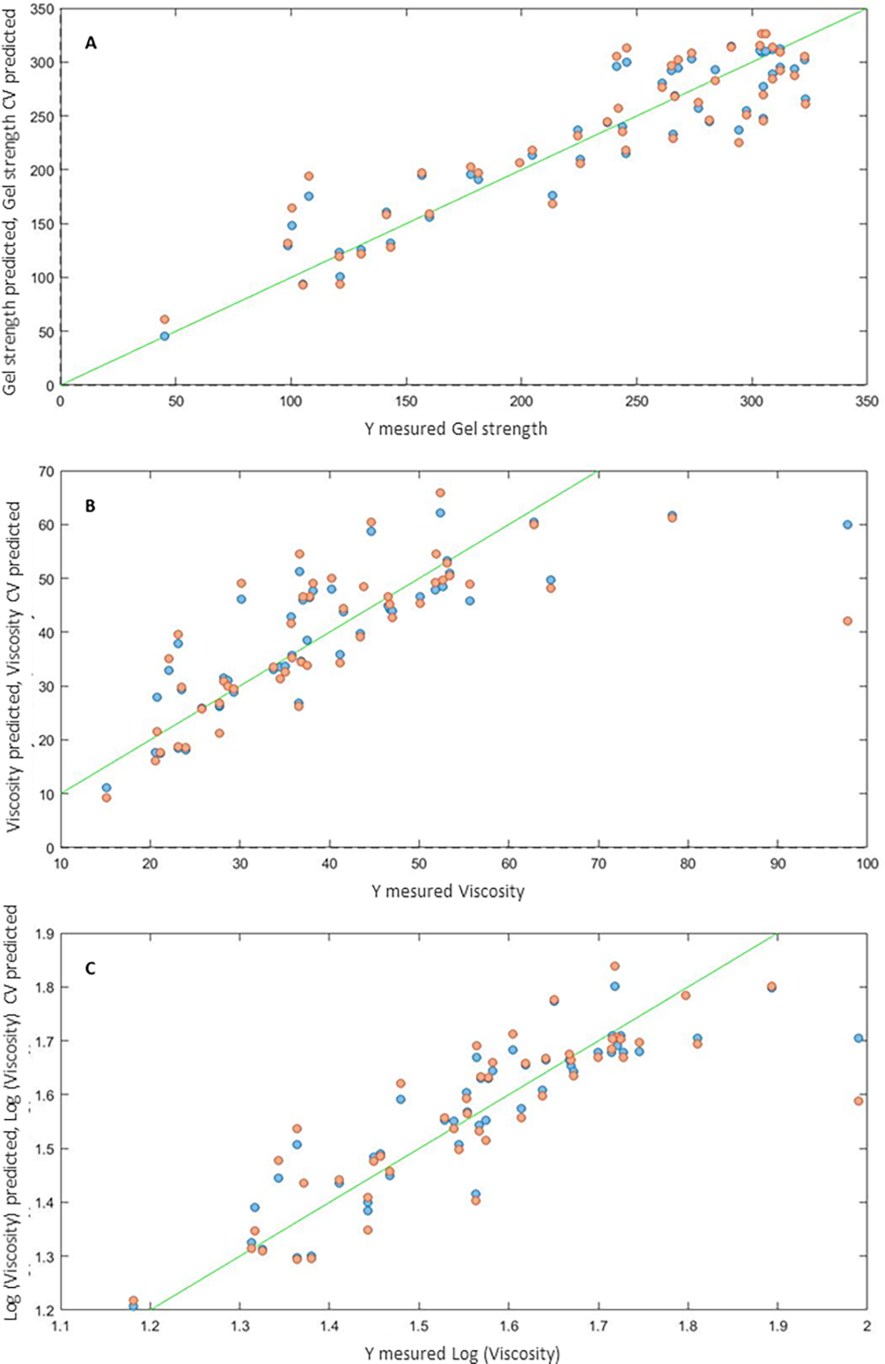
Correlation between predicted and measured values for gel strength (g) and viscosity (Pa.s). The blue points are the calibration dataset and the orange points are the cross validation.

The regression of physicochemical variables with respect to the latent variables, calculated by PLS and derived from the UV absorbance measured during elution, leads to the definition of very significant prediction models (see Fig 4 and Table 3). The molar distribution, as revealed by the graphs of UV absorbance versus elution time, thus reveals a lot about the technological properties of the gelatins. The correlations of the models determined here are much greater than those previously allowed by direct correlations with the mean values of the primary or polymeric chains.

**Table 3:** Assessment of prediction capacity by PLS regression of physicochemical properties (gel strength, viscosity) based on the UV absorbance in fractograms. The results lead to a model constructed from three latent variables.

	Calibration		Cross validation	
	r^2^cal	RMSEC	r^2^cv	RMSECV
*Gel force*	0.85	28.69	0.80	33.63
*Viscosity*	0.66	8.99	0.49	11.29
*Log (viscosity)*	0.79	0.074	0.69	0.091

The prediction of gel strength is better than that of viscosity, whether in calibration (r^2^cal = 0.85 vs r^2^call = 0.79) or cross validation (r^2^cv = 0.79 and r^2^cv = 0.69). This result may partly be explained by a loss of sensitivity in the baseline analysis for high viscosities, which leads to a nonproportional response (Fig 1B). These observations are consistent with those of Segtnan [34], who observed a less proportional response for high viscosities in models based on spectral measurements in near-infrared spectra. Therefore, the logarithmic transformation improves the calibration r^2^ from 0.66 for raw data to 0.79 for transformed data, and from 0.49 to 0.69 for cross validation (Fig 4C).

## Discussion

In manufacturing gelatin, collagen is denatured and loses its native structure. The structure of gelatin thus differs from that of collagen because of the partial reformation of the initial propeller structure. As indicated by the correlation coefficients, the distribution of molar mass varies greatly in commercial gelatins, which is attributed not only to the industrial treatment but also undoubtedly to the origin of the collagen [35]. In this study, a large set of different gelatin samples were tested by AsFlFFF to determine their gel strength and viscosity ranges.

A close relationship is found between the physicochemical properties, concentration, and the network structure that forms during gelling. In previous work (2,3), a low gelatin concentration (<1% w/w) was shown to promote the aggregation of gelatin chains into pelota, whereas for concentrations greater than 1%, gelatin tends to organize as a fibrillar nanostructure[36, 37]. These works studied the gel strength and gelatin viscosity at a concentration of 6.67% (w/w). At room temperature (20°C) and at these concentrations, gelatin forms a gel; that is, a protein network stabilized by weak bonds, which confers its viscoelastic properties[38].

The present work looks at the molar distribution of gelatin solutions at 0.2% (w/w), where gelatin does not form a structured network but instead undergoes gelatin gelling. The study of the radius of gyration highlights the nonlinear structure of gelatin in solution, which can be associated with the formation of pelota. In addition, the study of the radius of gyration reveals that the shape is independent of molar mass. These results are consistent with the work of *Guo et al*., which proposes a propeller structure with loop formation for gelatin in solution[39] which can correspond to the pelota observed under these conditions. We observe a molar mass that ranges from that of the trimer γ to over three orders of magnitude greater, which means that the polymeric chains must result either from the partial hydrolysis of collagen and are remnants of structures branched by covalent bonds, or from associations stabilized by weak bonds.

In addition, the polymers derived from the gelatin studied herein can attain a molar mass on the order of 1×10^7^g/mol, which is much greater (1×10^9^g/mol) than that observed under the same conditions by Rbii et al. (6) and Fraunhofer et al. (7). Although the distribution of molar mass found herein is consistent with their results, the gelatins are very different in terms of polymer chains (or aggregates). These differences in molar mass are the result of the different extraction processes used by Rbii et al. and Fraunhofer et al. In fact, some of the gelatins used in the present study correspond to different stages of the extraction sequence, which leads to a degradation of the physicochemical properties. The AsFlFFF technique differentiates between gelatin samples, mainly through the identification of high-molar-mass chains.

The analysis of correlations between gelatin fractions identified by AsFlFFF and the viscoelastic properties of the gels formed therefrom clearly shows the importance of polymer chains in the formation of gels with higher gel strength and viscosity.

## Conclusion

This work uses AsFlFFF to analyze the distribution of gelatin over its entire range of molar mass, which demonstrates that AsFlFFF is an interesting tool for studying the molar mass of hydrocolloids.

A chemometric approach based on the UV absorbance of pig-gelatin fractograms was used to determine the viability of predicting gel strength and viscosity from an integral fractogram. Combining the UV absorbance spectrum with a PLS regression analysis of spectral data gives very good predictions of the gel force (r^2^_Cal_ = 0.85) and the viscosity after logarithmic transformation of the data (r^2^_Cal_ = 0.79). The results highlight the importance of high-molar-mass molecules to explain the physicochemical properties of gels, without neglecting the influence of low-molar-mass chains.
